# Unintentional Child Injury in Rural and Urban Siddlagatta, Karnataka, India: A Community-Based Survey

**DOI:** 10.7759/cureus.47960

**Published:** 2023-10-30

**Authors:** Babitha Rajan, Shalini C Nooyi, Nanda Kumar B Sastry, Anjana George, Chandrika Rao, Sunilkumar B M

**Affiliations:** 1 Community Medicine, M.S. Ramaiah Medical College, Bangalore, IND; 2 Biostatistics, International Quality and Value Institute Advisors (IQVIA), Kerala, IND; 3 Pediatrics, M.S. Ramaiah Medical College, Bangalore, IND

**Keywords:** community based, urban, rural, siddlagatta, road traffic injuries, poisoning, falls, burns, animal-related injuries, unintentional childhood injuries

## Abstract

Introduction: Despite Indian children constituting approximately 31.4% of the population aged 0 to 14, a comprehensive exploration of childhood injuries within this demographic remains limited. This study aimed to examine the occurrence of unintentional injuries among children aged six months to 18 years in the Siddlagatta area and assess associated risk factors.

Methodology: A community-based, cross-sectional study on unintentional child injuries was carried out from March 2018 to September 2020 across 11 sites in India. Employing a two-stage cluster sampling method with probability proportionate to size, 2341 urban and rural families were selected from each site. Data on unintentional injuries sustained over the past 12 months were collected using the WHO child injury questionnaire, tailored and validated for the Indian context.

Results: The study encompassed 10,335 individuals in households, including 2695 children aged 6 months to 18 years. Among them, 309 children experienced 390 unintentional injuries in the preceding year, excluding minor incidents. A prevalence rate of 11.5% (95% CI: 10.3-12.7) was identified for unintentional injuries among children, excluding minor cases. Falls were the most prevalent injury type (183 cases, 53.8%), while poisoning incidents were the least frequent (one case, 0.2%). More than 50% of incidents occurred within domestic settings.

Conclusions: This study's outcomes underscore the prominence of fall-related injuries across all age groups and genders. Homes and schools emerged as primary settings for these injuries, highlighting the need for targeted preventive measures.

## Introduction

Injury accounts for a sizable portion of the burden on global health. More children are affected by child injuries than by all major diseases combined, which is a severe public health concern. Worldwide, unintentional injuries are considered a serious public health concern and the number one killer of children and adolescents [1-3]. Injury-related deaths account for more than 5.8 million annual fatalities, or 15,000 per day, making this a global health issue affecting both developed and developing nations [4].

Injury is defined as physical harm sustained due to an external force and energy transfer that exceeds a person's physiological tolerance and is brought on by unsafe conditions, actions, or environments [5]. Injuries can be unintentional, such as falls or drowning, and intentional, such as purposeful assault.

Roughly 950,000 children and adolescents under the age of 18 lose their lives to this tragic circumstance annually, solidifying its position as one of the primary contributors to youth mortality worldwide. Alarmingly, almost 90% of these instances are the result of unintentional injuries. Regrettably, this situation takes a particularly heavy toll on individuals aged 10 to 19, making it the leading cause of mortality within this age group [6]. Injuries played a significant role in accounting for 11.2% of disability-adjusted life years (DALYs), with a number of these injuries playing crucial roles in this overall impact. Among these, road traffic injuries stood out as a major contributor, constituting 27% of the total injury-related burden. The findings of the 2010 Global Burden of Disease study highlight that injuries collectively led to the loss of 278,665 DALYs across various age groups [7].

Accurately quantifying fatalities and injuries attributed to specific causes, as well as reliably estimating injury-related deaths in India, remains a complex challenge with no single definitive source. Based on data from the National Crime Records Bureau and a study conducted using available information, it is indicated that children contribute to approximately 10-15% of injury-related fatalities in India [8,9].

While some studies have explored unintentional child injuries using data from hospitals, there remains a significant gap in understanding the broader population-level factors that would offer a comprehensive view of the issue.

To address this gap and gain insights into the frequency and underlying causes of unintentional childhood injuries in India for children aged 6 months to 18 years, the Indian Council of Medical Research (ICMR) undertook the responsibility of commissioning this study.

## Materials and methods

Conducted between 2018 and 2020, the present study was initiated as an extramural project supported by the Indian Council of Medical Research (ICMR). This endeavour was part of a broader multicentric research initiative spanning 11 chosen locations meticulously selected by the funding body to ensure comprehensive representation across all regions of India [10]. The investigation unfolded as a community-based cross-sectional study from March 2018 to September 2020, specifically centred in the Karnataka region of the Siddlagatta taluka (a local term for a subdivision of a district).

Comprising an observational approach without any interventions, the study was executed with precision. The multicentric nature of the study involved carefully selecting diverse geographical regions across India to ensure a well-rounded representation. Coordination was established among the sites to maintain uniformity in protocols, training, operational procedures, and technological access. The study's participant pool encompassed children from urban and rural areas within Siddlagatta taluka, spanning the age spectrum from six months to 18 years.

The inclusion criteria for this study encompassed all children aged 6 months to 18 years, which included those who are 17 years and 11 months old, residing permanently in the study area for a minimum of six months. This category extends to visiting children and household help who have also been residing in the area for over six months. Additionally, the criteria include children within the same age range who have tragically passed away within the last year due to unintentional injuries.

Children with disabilities who were prone to injuries due to physical or mental impairment; children suffering from any severe communicable or non-communicable diseases; children whose parents refused to take part in the study; children with minor injuries; and children who were not permanent residents of the surveyed households were excluded from the study.

Sample size and sampling strategy

The determination of sample size adhered to the guidelines set forth by the World Health Organization (WHO) for conducting community surveys on injuries and violence [11]. This process took into consideration an overall childhood injury prevalence of 11.0%, accounting for minor injuries as well. A relative precision of 13% was factored in, along with a desired confidence level of 95%. The sample size was subsequently increased by 10% to accommodate potential non-responses. Moreover, the cluster sampling design was accounted for with a design effect of 2.

Drawing from the demographic distribution data between rural and urban areas as reported in the 2011 Census, a balanced approach guided the selection of 2341 households. This distribution was reflective of the institution's primary service area, determined by the district. Through a systematic process, a specific taluk was selected employing fundamental random sampling. The subsequent selection of households within each taluk was carried out using probability proportional to size (PPS) sampling for both urban and rural clusters. Aiming to match the workload capacity of four field workers visiting homes in a day, each cluster consisted of 16 households.

Data collection

At each site, a comprehensive workshop was convened to recruit and uniformly train a team of four medico-social workers, who would serve as field workers. This training spanned five days and effectively equipped these field researchers with essential skills. The workshop encompassed diverse aspects such as conducting an initial pilot study on child injuries, familiarizing with the assessment tool, cultivating relationships within the local community, mastering data collection techniques, and offering hands-on guidance for utilizing electronic handheld devices.

To ensure a systematic approach, homes were assigned sequential numbers, and field researchers crafted visual maps of each village in rural areas or localities in urban regions. Following an explanation of the study's objectives in the local language, informed written consent was procured from respondents, typically parents or primary caregivers. For children aged seven and above, verbal consent was sought directly from them.

**Figure 1:**
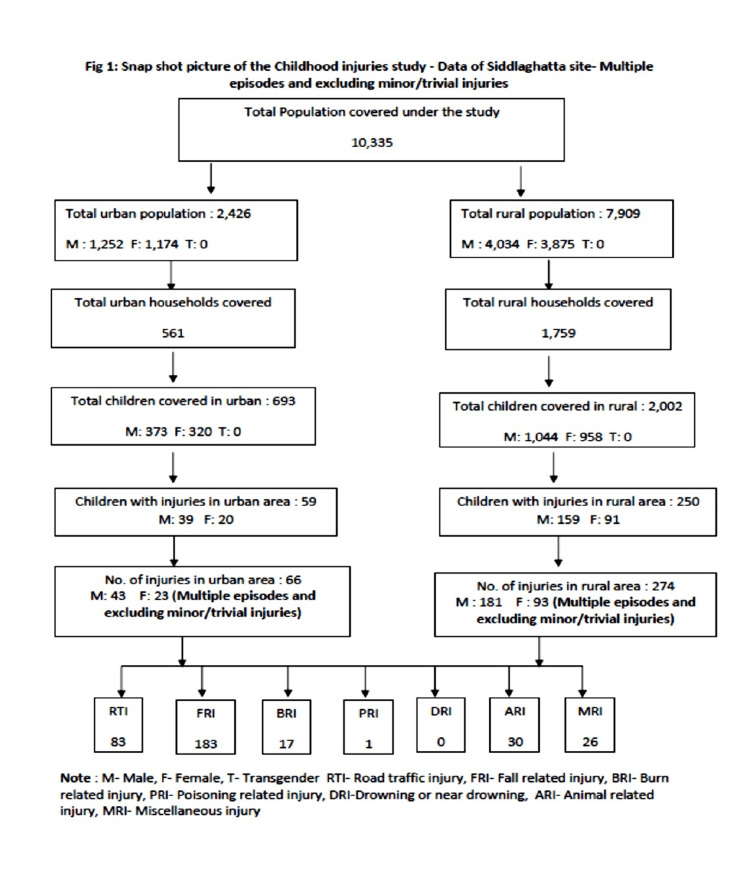
Flowchart of childhood injuries included in the study (data from the Siddlagatta site) This flowchart includes multiple episodes and excludes minor/trivial injuries. Abbreviations: M, male; F, female; T, transgender; RTI, road traffic injury; FRI, fall-related injury; BRI, burn-related injury; PRI, poisoning-related injury; DRI: drowning- or near drowning-related injury; ARI, animal-related injury; MRI, miscellaneous related injury

Interviews with children aged seven and older were undertaken not only to corroborate parental information but also to gather additional data pertaining to injuries experienced over the preceding three months. However, data concerning fatal injuries spanned a 12-month window and was cross-referenced with records maintained at the panchayat office (local term for village-level governing body). Post-interviews, caregivers were provided with verbal instructions on preventive measures and strategies to mitigate injuries.

The tool employed for data collection: The study employed the Child Injury Questionnaire formulated by the World Health Organization (WHO), tailored to suit the Indian context [11]. Adjustments were made to the definitions of injury types (including road traffic accidents, falls, burns, poisoning, drowning, and injuries caused by animals) and their severity (mild, moderate, and severe), adapting them from the established WHO standard definitions [12]. Any injuries falling outside of these predefined categories were classified as "various injuries."

The collected data underwent centralized analysis in accordance with an approved statistical analysis plan. A cloud-based application, developed by an external vendor and subjected to validation, was created for data entry. In-person interviews yielded data that was seamlessly inputted into the central database through handheld devices. Each centre's field workers were equipped with four handheld devices, and an additional device was designated for the supervisor. The software was thoughtfully designed with data quality assurance in mind, incorporating features like valid entries, skip logic, mandatory fields, and consistency checks. The web-based platform facilitated real-time access to unique electronic data for each site, with the capability to download copies, thanks to a robust management information system and an advanced dashboard.

A robust quality control framework was established. The coordinating centre compiled an operation manual and a training manual, disseminating them to all field personnel across participating sites to ensure consistency. Monthly virtual meetings were organized between the central and national coordinating sites, along with other site investigators and field workers, utilizing the Internet as a platform for discussion. These sessions served as avenues for centralized monitoring and guidance, maintaining data quality. The real-time dashboard enabled continuous monitoring of data across sites. Additionally, to ensure data accuracy, investigators at each site independently re-visited 5% of randomly selected households, cross-verifying the data and resolving any discrepancies.

Statistics

Data analysis was conducted using the Statistical Package for the Social Sciences v. 18.0 (SPSS Inc., Chicago, IL). The severity of injuries served as the basis for data categorization. Notably, minor injuries were excluded from the subsequent estimations.

To assess the relationships between factors such as prevalence rate, age, gender, and other variables, statistical significance was determined utilizing either the chi-square test or Fisher's exact test. For assessing the significance of mean value differences between the two groups, the Student's t-test was employed. The threshold for establishing statistical significance was set at a probability value of less than 0.05.

Prevalence rates, calculated for a three-month period, were supplemented with 95% confidence intervals, affording a robust representation of the data's precision.

Operational definitions

Operational definitions have been established to clarify the categorization of injuries within this study:

(1) A minor or trivial injury is defined as an injury that does not necessitate immediate medical attention, can be managed at home, does not result in absenteeism from work or school, and does not impede the performance of daily activities; (2) a moderate injury is characterized by the need for medical attention, resulting in one to three days of absence from work or school, or causing a temporary incapacity to carry out routine daily activities; (3) a major injury refers to injuries that require hospitalization for less than 10 days - this definition encompasses accidents demanding substantial medical intervention but not necessarily surgical procedures; (4) a serious injury is defined as an injury requiring ten or more days of hospitalization or a significant surgical intervention; (5) a severe injury results in permanent disability, which may include loss of sight, hearing, limb, mobility, or mental faculties - emotional and psychiatric causes were excluded due to challenges in diagnosis and classification; (6) a fatal injury leads to death, whether immediate or delayed.

The types of injuries included in this study encompass road traffic accidents, falls, burns, poisoning, drowning, animal-related injuries, and other minor or trivial injuries.

## Results

In the Siddlagatta region, it was ascertained that 11.5% of children experienced injuries, with a 95% confidence interval ranging from 10.3% to 12.7%. Among these, male children exhibited a higher injury prevalence at 13.9%, while female children recorded an injury prevalence of 8.7%. This disparity between the sexes yielded a statistically significant difference (p < 0.001), with the overall injury prevalence standing at 12.6% (95% CI: 11.3-13.9).

Furthermore, the analysis unveiled that injuries were more prevalent in rural areas (12.5%) compared to urban areas (8.5%). The divergence in injury prevalence based on the area of residence was statistically significant (p < 0.001) (Table 1).

**Table 1:** Socio-demographic characteristics of the study subjects of the Siddlagatta region (N= 2695) *375 respondents declined to reveal their economic status.

Variables	Number (N=2695)	No. with injury n=309	Prevalence(95% CI)
Age group			
6 months - < 1 year	70	0	-
1-4 years	548	55	10.0(7.6-12.9)
5-9 years	827	105	12.7(10.5-15.2)
10-14 years	776	103	13.7(10.9-15.9)
15-<18 years	474	46	9.7(7.1-12.7)
Gender			
Male	1417	198	13.9(12.2-15.9)
Female	1278	111	8.7(7.2-10.4)
Area of residence			
Urban	693	59	8.5(6.5-10.8)
Rural	2002	250	12.5(11.0-14.0)
Type of family			
Nuclear family	1623	193	11.8(10.3-13.6)
Joint family	380	28	7.3(4.9-10.5)
Three-generation family	692	88	12.7(10.3-15.4)
Religion			
Hindu	2193	277	12.6(11.2-14.0)
Muslim	500	32	6.4(4.4-8.9)
Christian	2	0	-
Socio-economic status (modified Kuppuswamy scale)	N=2,320*		
Upper (26-29)	2	0	-
Upper middle (16-25)	31	3	9.6(2.0-25.7)
Upper lower (11-15)	1512	202	13.3(11.6-15.2)
Lower middle (5-10)	339	27	7.9(5.3-11.3)
Lower (01-04)	436	41	8.7(6.2-11.8)

Among the various injury types, road traffic injuries (RTIs) accounted for 3.1% of cases (95% CI: 2.4-3.8), followed closely by fall-related injuries at 6.8% (95% CI: 5.8-7.8). These two categories were identified as the most frequent contributors among the 309 injured children. Other types of injuries exhibited lower prevalence rates. Notably, no injuries related to drowning were reported (Table 2).

**Table 2:** Prevalence rate (%) of unintentional injuries by type of injuries

Type of injury	Number of injuries	Prevalence	95% confidence interval
Road traffic injury	83	3.1	2.4 - 3.8
Falls	183	6.8	5.8 - 7.8
Burns	17	0.6	0.03 - 1.0
Poisoning	1	0.04	0.0 - 0.2
Animal related	30	1.1	0.7 - 1.5
Miscellaneous	26	1.0	0.6 - 1.4
Total	340	12.6	11.3 -13.9

In contrast to the 111 instances among female children, a total of 224 diverse types of injuries were reported among the 198 injured male children. A breakdown of gender-specific prevalence rates for various injury types can be found in Table 3. Notably, falls emerged as the most frequent cause of injuries for both male children (7.1%) and female children (5.4%). The distinction in fall prevalence rates between the two genders was statistically significant (p = 0.01). Similar findings were observed for road traffic injuries (RTIs). Burn injuries exhibited a relatively common occurrence, with both sexes displaying similar prevalence. Due to their low prevalence rates, comparisons involving other injury types could not be conclusively drawn.

**Table 3:** Prevalence rate (%) of types of injuries among the study subjects by gender 95% CI (confidence interval)

Gender (M: 1417; F: 1278)	Types of injuries							
	Road traffic injury	Falls	Burns	Poisoning	Drowning	Animal related	Miscellaneous	Total
Males (224 injuries)	4.5	7.1	0.6	0	0	1.5	1.1	15.8 (Prevalence of all types of injuries)
95% CI	3.5-5.7	5.8-8.6	0.2-1.2	-	-	0.9-2.2	0.6-1.8	13.9-17.8
Females (116 injuries)	1.2	5.4	0.6	0.1	0	0.7	0.8	9.1 (Prevalence of all types of injuries)
95% CI	0.6-1.9	4.2-6.8	0.2-1.2	0.0-0.4	0	0.3-1.3	0.3-1.4	7.5-10.8
P-value	<0.001	0.01	0.83	-	-	0.04	0.25	<0.001

In terms of injury severity, 131 out of the total 340 injuries (38.5%) were categorized as moderate, followed by major injuries at 89 instances (26.2%). Nearly equal proportions of injuries fell into the serious or severe categories, accounting for 16.5% and 18.8%, respectively. A more substantial percentage of children who suffered falls (46.9%) experienced moderate injuries, while 34.9% of those with road traffic injuries endured major ones. Burn injuries posed a severe threat, as most cases resulted in serious to severe damage (Table 4).

**Table 4:** Distribution of injuries among study subjects according to the severity of injury (Including multiple injuries and multiple episodes)

Severity	Road traffic injury; n (%)	Falls; n (%)	Burns; n (%)	Poisoning; n (%)	Animal related; n (%)	Miscellaneous; n (%)	Total; n (%)
Severe injury	14 (16.9)	36 (19.7)	5 (29.4)	0 (0.0)	2 (6.7)	7 (26.9)	64 (18.8)
Serious injury	17 (20.5)	26 (14.2)	5 (29.4)	0 (0.0)	4 (13.3)	4 (15.4)	56 (16.5)
Major injury	29 (34.9)	35 (19.1)	5 (29.4)	0 (0.0)	14 (46.7)	6 (23.1)	89 (26.2)
Moderate injury	23 (27.7)	86 (46.9)	2 (11.8)	1 (100.0)	10 (33.3)	9 (34.6)	131 (38.5)
Total	83 (100.0)	183 (100.0)	17 (100.0	1 (100.0)	30 (100.0)	26 (100.0)	340 (100.0)

The prevalence of childhood injuries among males residing in rural areas exceeded that of females, with rates of 15.2% for males and 9.5% for females. On average, a child experienced injuries around 1.0 to 1.2 times per year, as indicated in Table 5.

**Table 5:** Prevalence rate (%) of unintentional childhood injury based on gender and area of residence *Considering at least one injury per child.

	Urban		Rural		Total	
	Male	Female	Male	Female	Male	Female
Prevalence rate (%) of children with injury*	10.5	6.3	15.2	9.5	13.9	8.7
95% confidence interval	7.5-14.0	3.8-9.5	13.1-17.6	7.7-11.5	12.2-15.9	7.2-10.4
Average number of injuries per injured child	1.1	1.2	1.1	1.0	1.1	1.1

Considering falls as the predominant cause of childhood injuries in this study, it was observed that among the 183 children who sustained falls, a higher percentage had moderate injuries. Specifically, 44 males (40%) and 42 females (57.5%) fell into this category. These higher percentages of children affected were primarily within the age range of 5-9 years, with 20 males (45%) and 13 females (30.9%) representing this group. Additionally, children aged 1-4 years constituted the majority of those injured at home, encompassing 42 males (38.2%) and 38 females (52.1%). The distribution of injuries was nearly equal for school-related (21% to 23% for males and 20% to 21% for females) and road-related incidents.

The home environment emerged as a prominent setting for injuries in children, particularly for falls and burns; additionally, over 80% of the injuries caused by animals occurred at home. Schools and roads are also common places where children experience injuries, with boys frequently encountering mishaps on playgrounds. Among the physical manifestations of injuries, fractures and open wounds rank as the most prevalent.

When seeking medical care, children who suffered injuries from falls and animal-related incidents exhibited a higher tendency to seek treatment at government hospitals as opposed to private clinics or nursing homes. Remarkably, about 30% of injured children who sought medical attention arrived at healthcare facilities within an hour of the accident. In cases of road traffic injuries (RTIs) or burns, children typically sought treatment at private clinics or nursing homes.

Nearly half of the injured children received some form of first aid, with a relatively higher proportion coming from rural areas.

## Discussion

The findings of this study shed light on the prevalence and key factors associated with unintentional childhood injuries in the Siddlaghatta region, offering valuable insights into this important public health concern. Childhood injuries have been a subject of concern worldwide, and their prevalence can vary significantly across regions and populations. Several studies conducted in various parts of India have reported a wide range of injury prevalence rates, spanning from 11% to 64% [13-15]. In the Siddlagatta region, it was ascertained that 11.5% of children experienced injuries, with a 95% confidence interval ranging from 10.3% to 12.7%. Among these, male children exhibited a higher injury prevalence at 13.9%, while female children recorded an injury prevalence of 8.7%. This disparity between the sexes yielded a statistically significant difference (p < 0.001), with the overall injury prevalence standing at 12.6% (95% CI: 11.3-13.9). The wide range in prevalence can be attributed to disparities in data sources, definitions, and selection criteria used to gauge the burden of injuries. This figure reflects the substantial burden of childhood injuries in this region and underscores the need for targeted preventive measures. Some factors contributing to childhood injuries may be non-modifiable, such as genetic predispositions or natural environmental conditions, making it challenging to identify actionable interventions.

One noteworthy observation from our study is the significant gender disparity in injury rates, with boys experiencing a higher prevalence of injuries compared to girls, at a ratio of 2:1. This finding is consistent with cultural norms and patterns where boys are often encouraged to engage in more outdoor activities and play compared to girls, particularly in the age group of 5-14 years. This gender-based difference in injury rates has also been reported in previous studies [13,14]. However, it is important to note that not all studies have found such a strong association between gender and injury prevalence, as evidenced by a study conducted in Agartala [15]. This suggests that the relationship between gender and childhood injuries may be influenced by regional and cultural factors and highlights the complexity of this issue.

In conclusion, this study provides valuable insights into the prevalence, gender disparities, injury types, and contributing factors associated with unintentional childhood injuries in the Siddlaghatta region. The high prevalence of injuries, particularly falls and road traffic injuries, underscores the urgency of implementing preventive measures and safety initiatives. Additionally, the observed gender disparity in injury rates highlights the need for gender-sensitive strategies to address childhood injuries effectively. These findings contribute to a broader understanding of the landscape of childhood injuries in Siddlaghatta and provide a foundation for future research and public health interventions aimed at reducing childhood injuries in this community.

Falls emerged as the most prevalent type of injury among children in Siddlaghatta, a finding consistent with the global pattern where falls are a leading cause of pediatric injuries, accounting for up to 52% of injury cases seen in emergency rooms, and even higher prevalence in Asia, at 43% [8,13,16-18]. In our study, falls were identified as the second most common cause of injuries, following RTIs, which constituted 15.9% of all injuries. This underscores the importance of addressing fall prevention strategies, especially within the home environment, to reduce the burden of childhood injuries in the Siddlaghatta region.

In our study, cuts, bites, and open wounds were found to be the most frequent injury types, making up around 23.9% of all injuries. Burns, bruises, and superficial injuries constituted 15% of injuries, while fractures accounted for 19%. These patterns align with data from similar studies [13,19,20], highlighting the commonality of injury types among children. Understanding the specific injury types and their prevalence can inform targeted interventions and educational programs aimed at reducing the occurrence of these injuries [19,20].

Limitations

Notwithstanding the study's credibility, it is crucial to recognize and address certain inherent limitations in its design. The cross-sectional nature of this study posed certain challenges, primarily in its ability to monitor the trajectory of injured children over time and pinpoint the exact causal factors behind their injuries. Employing a retrospective data collection approach also introduced the potential for underestimating injury incidence, a challenge frequently encountered in community-based surveys. Importantly, it is essential to acknowledge that this limitation is not unique to our study but is a common constraint shared by similar research efforts in the field. Also, as this study is a cross-sectional study, the probability of recall bias may lead to data inaccuracies when parents or caregivers find it challenging to recollect events related to childhood injuries.

Despite these acknowledged constraints, the findings of this study offer valuable insights into the prevalence and dynamics of childhood injuries in the Siddlaghatta region. The study's ability to shed light on the burden of childhood injuries, gender disparities in injury rates, prevalent injury types, and contributing factors provides a foundational understanding of this critical public health issue within the local context. While limitations exist, these findings still serve as a valuable resource for informing future research and public health initiatives aimed at reducing childhood injuries in the Siddlaghatta community.

## Conclusions

It was evident from the present study that fall-related injuries were commonly reported among children across all age groups and genders, and the most common place of injuries was at home, followed by school. Studies can be conducted that are directed at finding appropriate interventions that are effective in reducing injuries at home and school. Some factors contributing to childhood injuries may be non-modifiable, such as genetic predispositions or natural environmental conditions, making it challenging to identify actionable interventions. Unintentional childhood injuries may have complex and multifactorial causes that unfold over time. A cross-sectional study captures data at a single point, making it challenging to establish causal relationships or identify temporal trends.

To address this issue effectively, rallying support for dedicated injury prevention research teams is essential. By channelling efforts into robust analytical research methods, it becomes possible to identify the most effective strategies for expanding and fortifying the knowledge foundation that informs injury prevention policies and practices. This approach empowers the development of informed measures to safeguard children from preventable harm and create safer environments for their growth and development.
